# Traumatic Lateral Abdominal Wall Hernia: A Rare Manifestation of Blunt Trauma

**DOI:** 10.7759/cureus.37534

**Published:** 2023-04-13

**Authors:** Ioannis D Passos, Athanasios Katsaounis, Aristoklis Paraschou, Georgios E Papavasileiou, Apostolos Galatas, Isaak Kesisoglou

**Affiliations:** 1 Surgical Department, 219 Mobile Army Surgical Hospital, Didymoteichon, GRC; 2 3rd University Surgical Department, AHEPA University General Hospital, Aristotle University of Thessaloniki, Thessaloniki, GRC

**Keywords:** lateral, incarceration, tension free mesh repair, blunt force trauma, mesh repair, traumatic abdominal wall hernia

## Abstract

Traumatic abdominal wall hernia (TAWH) following blunt injury is a rare clinical entity, induced by traumatic disruption of the abdominal wall's muscle and fascia, alongside abdominal organ herniation. A thorough clinical examination and a high level of suspicion are necessary for the diagnosis. We present the case of a 45-year-old individual who presented to the surgical outpatient clinic with a left lateral bulge in his belly caused by a mountaineering accident. After obtaining a thorough history of the mechanism of injury and clinical assessment, abdominal ultrasonography and computed tomography (CT) scan revealed a significant traumatic left lateral abdominal wall hernia. The patient subsequently underwent an open surgical mesh repair, followed by anatomical and functional restoration of the muscular deficit over the mesh, with an uneventful postoperative course. TAWH constitutes a diagnostic challenge, and in many cases remains untreated for long periods of time. Considering that TAWH occurs in less than 1% of all blunt abdominal trauma, many surgeons are unaware of this rare manifestation. Here we suggest that elective surgery with an open, tension-free polypropylene mesh repair appears to be an appropriate therapeutic option.

## Introduction

Traumatic abdominal wall hernia (TAWH) has been described in the literature for over a century, with Selby being the first to report such a case in 1906 ​[1]. TAWH is considered a rare clinical entity induced by disruption of the abdominal wall musculature and fascia, while the skin remains intact, as a result of blunt abdominal trauma such as deceleration injury from a seat belt ​[2]. While it can occur anywhere in the abdomen, it is most frequently observed in the lower and lateral quadrants. The prevalence of hernia in patients with blunt trauma is approximately 1% ​[2-4]. Since no definitive diagnostic criteria or official classification for TAWH currently exist ​[2,5], the ideal timing and course of treatment are subject to debate. In this particular study, we present a case of TAWH and our surgical approach. 

## Case presentation

**Figure 1:**
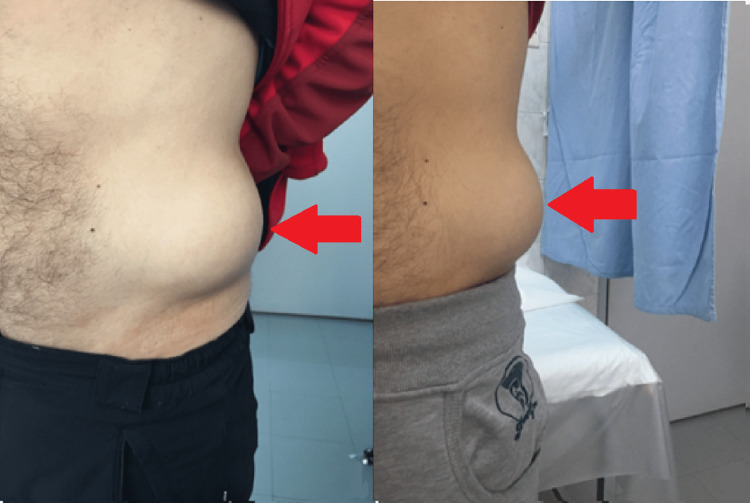
The traumatic left lateral abdominal wall hernia of the patient

**Figure 2:**
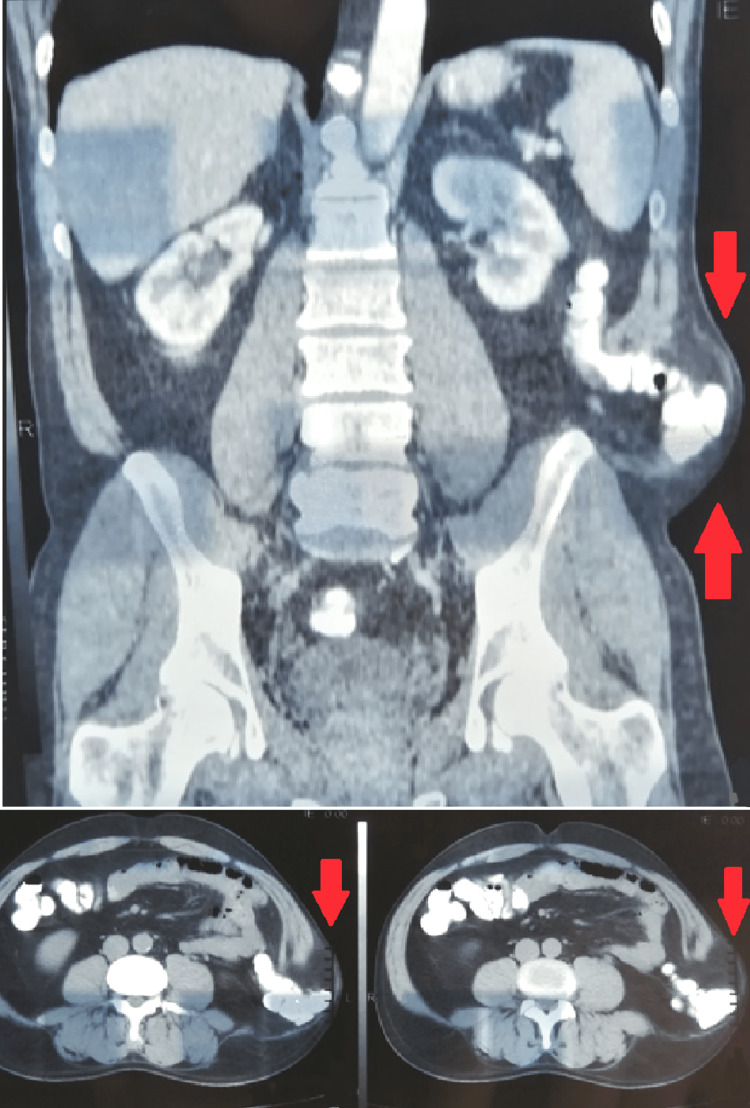
Abdominal computed tomography scan CT scan of the abdomen revealing the presence of a defect in the left lateral abdominal wall, which was 9 cm in diameter, and a hernia sac containing loops of the large bowel.

A 45-year-old man presented to our surgical outpatient clinic complaining about an immense left lateral mass in his abdomen, induced by falling onto a boulder while mountaineering a few months ago (Figure 1). After a complete history of the mechanism of injury was obtained, the clinical assessment revealed a 6-7 cm, bulging, round mass that was painless and, on palpation, was firmly attached and well demarcated. Ultrasound scan (U/S) of the soft tissues of the lower abdomen revealed the loss of continuity of the left lateral abdominal wall with the presence of a hernia that contained loops of the large bowel without evidence of strangulation. An abdominal computed tomography (CT) scan revealed the presence of a defect in the left lateral abdominal wall which was 9 cm in diameter and a hernia sac that contained loops of the large bowel (Figure 2). The defect started at the level of the quadratus lumborum muscle, causing an anterior/medial shift of the lateral abdominal wall musculature. We concluded that the patient suffered from a large traumatic left lateral abdominal wall hernia.

**Figure 3:**
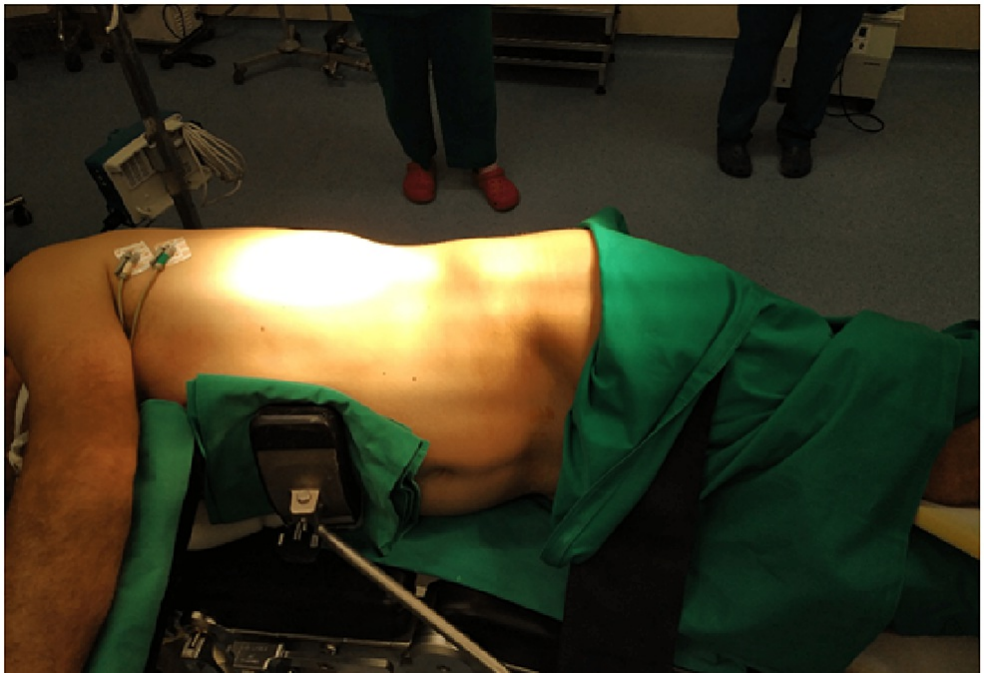
Left lateral decubitus position of the patient in the operating room.

**Figure 4:**
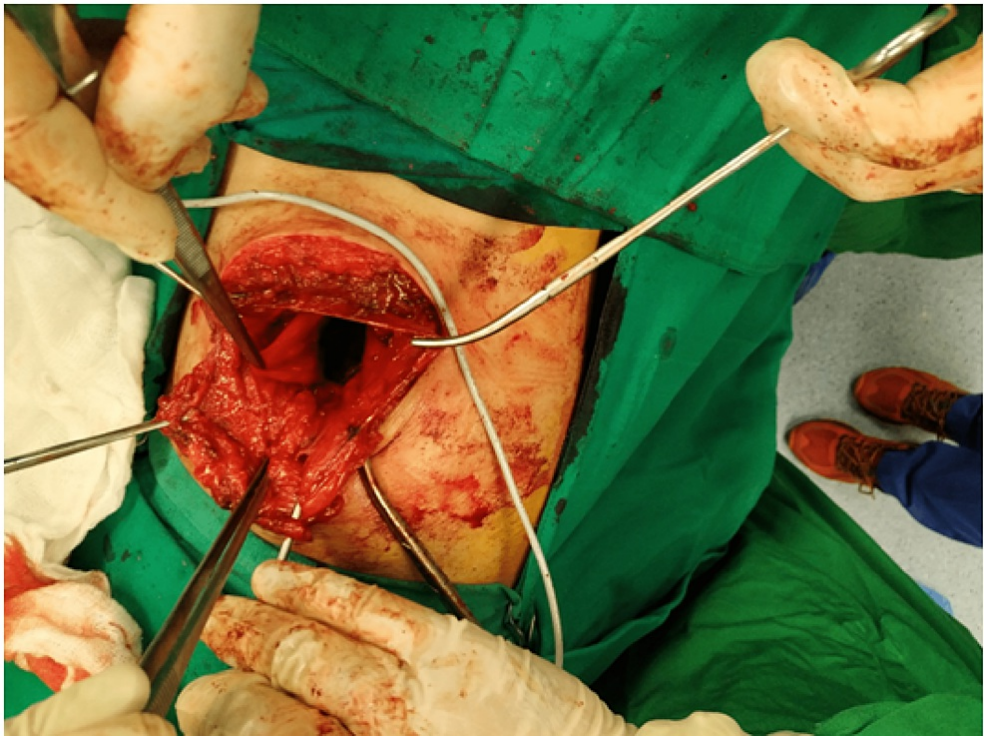
Dissection of the hernia sac and reduction back to the peritoneal cavity.

**Figure 5:**
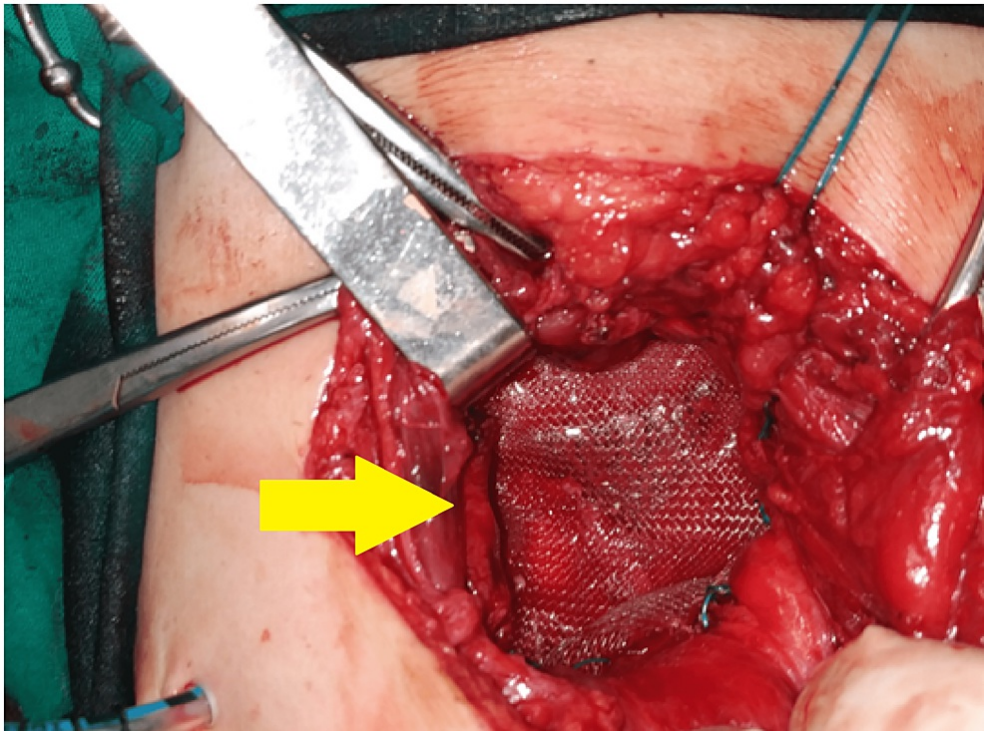
Mesh placement over the defect and below the muscles.

**Figure 6:**
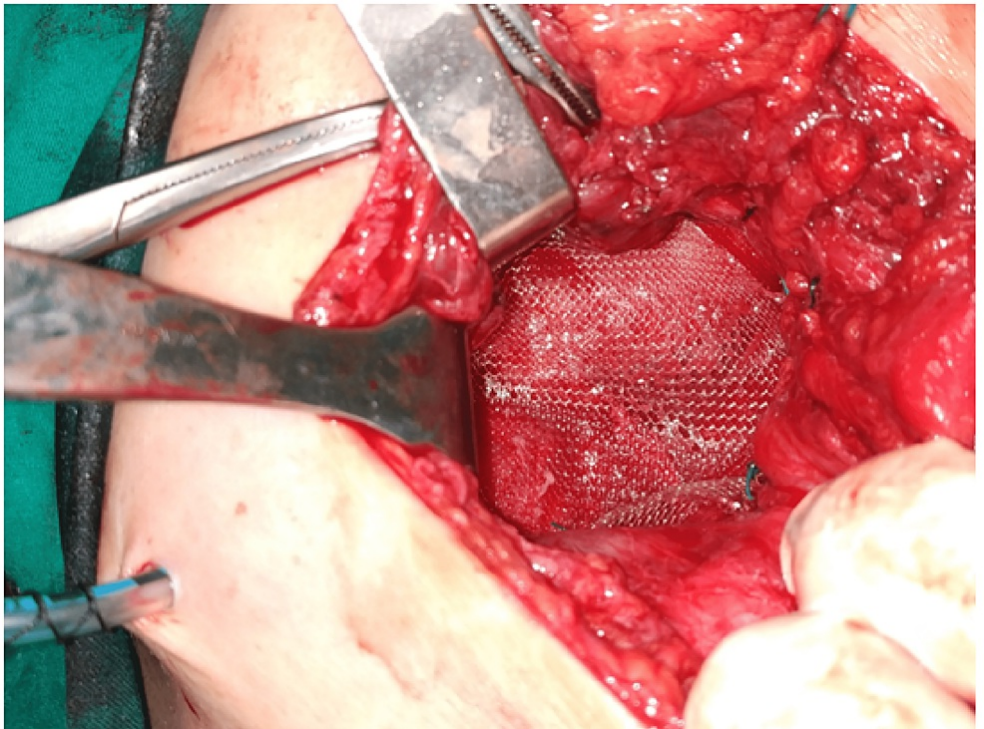
Stabilization of the mesh through sutures in the iliac crest, the thoracolumbar fascia, the transversus abdominis, and the posterior lamina of the rectus abdominis sheath.

**Figure 7:**
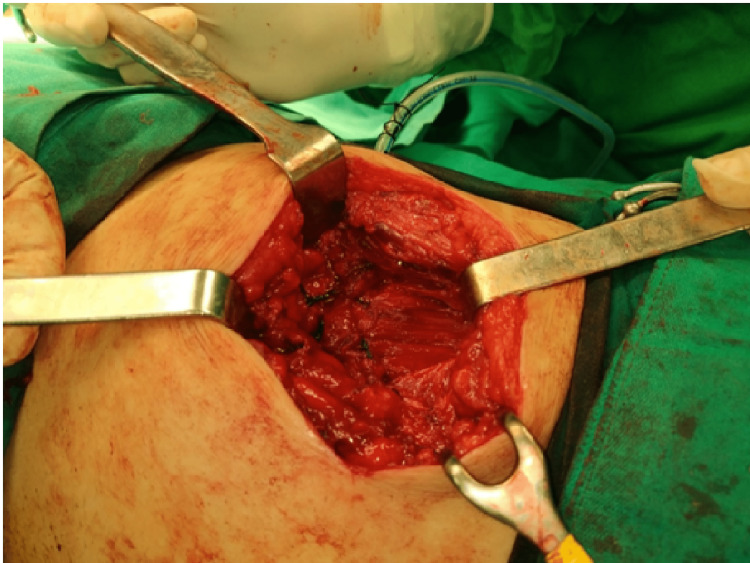
Suturing of the disrupted muscles over the mesh placement.

**Figure 8:**
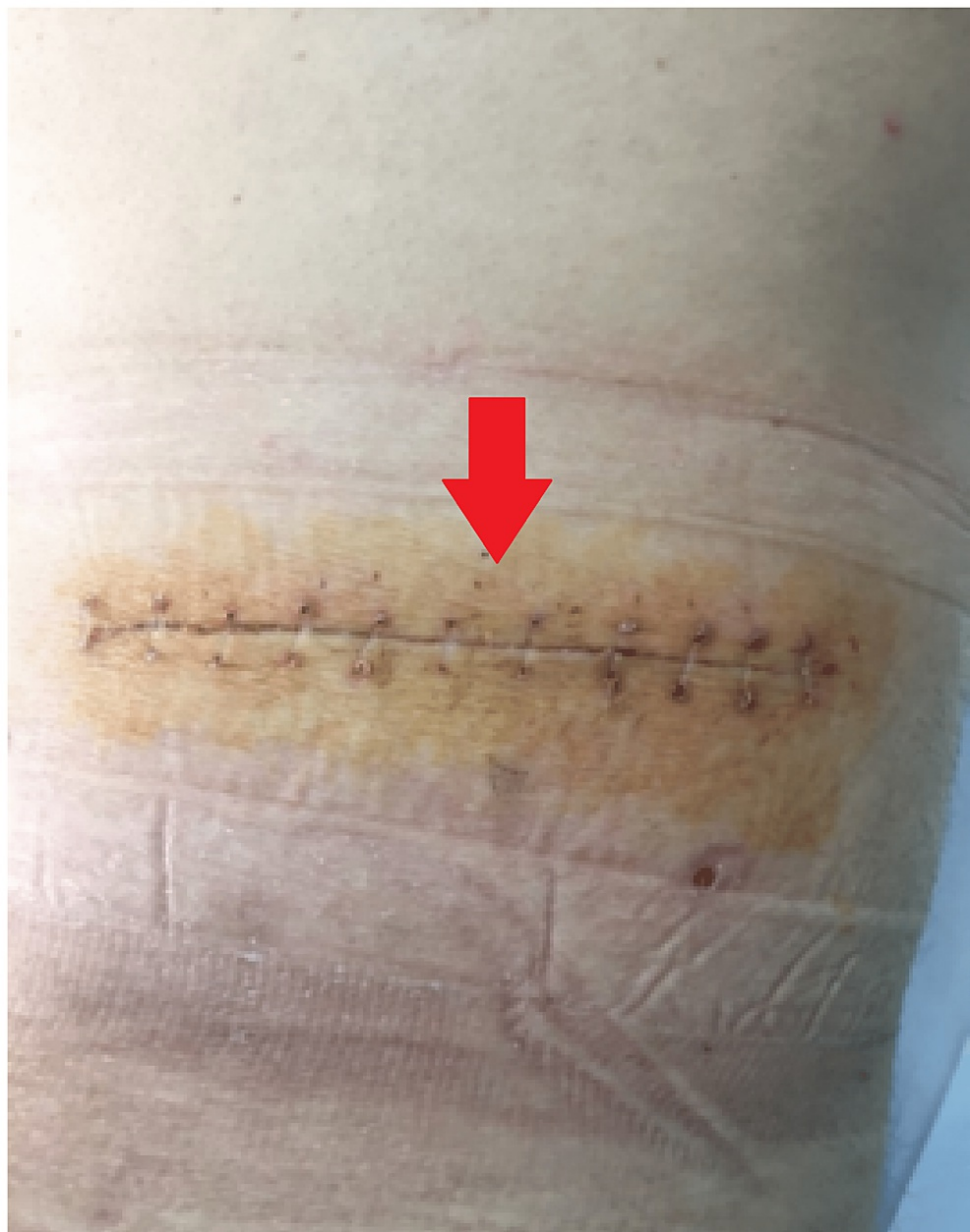
Suture removal on the 12th postoperative day.

The patient underwent elective open surgical mesh repair. He was placed in a left lateral decubitus (nephrectomy) position and a transverse incision over the hernia protrusion was made (Figure 3). A complete traumatic detachment of the origin of the three lateral abdominal muscles from the iliac crest was ascertained. The hernia sac was found and reduced into the peritoneal cavity, where a polypropylene mesh was placed over the defect and stabilized through sutures in the iliac crest, the thoracolumbar fascia, the transversus abdominis, and the posterior lamina of the rectus abdominis sheath (Figures 4-6). The disrupted muscles were sutured over the mesh (Figure 7). Apart from a small seroma formation at the incision site, which was treated conservatively and resolved, the patient's postoperative course was uneventful, with rapid onset of intestinal activity, food tolerance, and mobilization within hours of surgery. He was discharged on the fifth postoperative day, as we opted to enable him to be completely painkiller independent. On the 12th postoperative day, the sutures were removed (Figure 8).

 

## Discussion

TAWH is considered a rare sequela of blunt trauma with only a few cases having been described in the current literature. This infrequent type of hernia is defined by disruption of the abdominal wall musculature while the skin remains intact ​[6]. A proposed classification by Wood et al. divides TAWH into three subtypes: (a) small defects caused by impact against blunt objects (i.e. handlebar hernia), (b) bigger defects caused by motor vehicle injuries, and (c) injuries caused by deceleration forces [7]. 

TAWH is more prevalent in younger individuals, usually under the age of 50, since they're more likely to be involved in automobile accidents. A thorough history and clinical examination are essential in the diagnosis of TAWH. Abdominal discomfort, abrasions, ecchymosis, or hematoma may be the only clinical findings, while the differential diagnosis should always include pre-existing hernias, abdominal wall hematomas, and tumors ​[8]. Nonetheless, since the clinical presentations of these patients vary, it is probable that the abdominal muscle disturbance and any concomitant intra-abdominal injuries are not evident. In some cases, a palpable mass or defect that can be reduced into the peritoneal cavity may be identified. Late presentation of this situation includes incarceration of bowel loop, strangulation, perforation, and signs of peritonitis. CT scan is the imaging modality of choice for both establishing the diagnosis of TAWH and classifying the hernia since it is helpful in defining the anatomy of the defect as well as evaluating concomitant injuries ​[9]. A plain oblique or lateral abdominal X-ray may also aid in the diagnosis, revealing bowel loops outside the peritoneal cavity ​[6,10]. 

Early diagnosis and surgical intervention for TAWH are critical aspects to the outcome of these patients as they minimize the risk of entrapment of the bowel in the defect, which in turn can lead to strangulation or perforation. Surgical exploration, typically through a midline incision, aids in the identification of related injuries or those missed by the CT scan and allows for prompt treatment [10]. Individuals with minor defects may be treated without the use of prostheses, but those with major muscular abnormalities or those whose surgeries have been postponed will require mesh repair ​[8]. A number of studies have shown that primary TAWH repair increases both surgical site infection and recurrence rates up to 37.5% ​[11,12]. Emerging data have demonstrated that patients can be safely followed and have their TAWH repaired electively at two to three months after injury. A recently published algorithm for TAWH management has favored primary repair for anterior TAWH or for patients requiring an urgent laparotomy for other trauma-related causes, and elective repair for lumbar or lateral TAWH that do not require an urgent laparotomy ​[13]. However, TAWH is still associated with high morbidity and mortality, attributed in part to misdiagnosed cases ​[14]. 

The surgical treatment of TAWH includes the primary closure of the defect in layers, with or without the use of a prosthetic mesh repair. When a tension-free repair is possible and there is no underlying hollow viscus injury, the mesh may be employed as an on-lay graft (as in our case), below or over the sutured repair. Laparoscopic repair of TAWH has also been reported as it utilizes the advantages of laparoscopy in preventing negative laparotomies and revealing any underlying condition ​[15]. 

In our case, the choice of an open transverse incision over the hernia protrusion was made in order to achieve better access to the hernia sac and the disrupted musculature of the left lateral abdominal wall. There was no underlying hollow viscus injury and thus a tension-free repair of the defect with the use of a prosthetic polypropylene was performed. In addition, an anatomic restoration of the muscular deficit over the mesh was established, by suture fixation of the origin of the three lateral abdominal muscles on the periosteum of the iliac crest (in their natural anatomic location). The above-mentioned approach was utilized to achieve functional abdominal wall restoration. 

## Conclusions

In trauma patients, TAWH is a rare clinical entity that requires a high level of clinical suspicion. In addition to a thorough clinical assessment, CT appears to be the gold standard for confirming the diagnosis, screening for concomitant intra-abdominal injuries, and classifying the hernia. In terms of surgical treatment, both an open surgical approach via a transverse incision and laparoscopic repair of TAWH are viable options. Nevertheless, since TAWH are associated with significant morbidity and mortality, the timing of diagnosis and surgical intervention determines the outcome of these patients.
